# Multi-Antibiotic Resistance and Factors Affecting Carriage of Extended Spectrum β-Lactamase-Producing Enterobacteriaceae in Pediatric Population of Enugu Metropolis, Nigeria

**DOI:** 10.3390/medsci7110104

**Published:** 2019-11-17

**Authors:** Angus N. Oli, Vitalis I. Ogbuagu, Chika P. Ejikeugwu, Ifeanyichukwu R. Iroha, Malachy C. Ugwu, Chijioke M. Ofomata, Kenneth N. Okeke, George O. Emechebe, Jude C. Okoro, Chukwudi O. Okani, Stanley K. Onah

**Affiliations:** 1 Department of Pharmaceutical Microbiology and Biotechnology, Faculty of Pharmaceutical Sciences, Nnamdi Azikiwe University, Awka, P.M.B 5025, Anambra State, Nigeria; vitalisogbuagu@yahoo.com (V.I.O.); mc.ugwu@unizik.edu.ng (M.C.U.); 2 Department of Applied Microbiology, Faculty of Science, Ebonyi State University, Abakaliki, P.M.B 053, Ebonyi State, Nigeria; ejikeugwu_chika@yahoo.com (C.P.E.); iriroha@yahoo.com (I.R.I.); 3 Department of Clinical Pharmacy and Pharmacy Management, Faculty of Pharmaceutical Sciences, Agulu, Nnamdi Azikiwe University, Awka, P.M.B 5025, Anambra State, Nigeria; chijiokeofomata@gmail.com; 4 Department of Paediatrics, College of Health Sciences, Faculty of Medicine, Nnamdi Azikiwe University, Nnewi Campus, Nnewi 435101, Anambra State, Nigeria: kenwados01@yahoo.co.uk (K.N.O.); onahstan@gmail.com (S.K.O.); 5 Department of Pediatrics, Faculty of Clinical Medicine, Chukwuemeka Odumegwu Ojukwu University, Awka Campus, Awka 420108, Anambra State, Nigeria; nnabuike20g@yahoo.com; 6 Department of Paediatrics, Imo State University Teaching Hospital Orlu 473271, Imo State, Nigeria; jubhildr@yahoo.com; 7 Department of Histopathology, Faculty of Clinical Medicine, Chukwuemeka Odumegwu Ojukwu University Teaching Hospital Amaku, Awka, 420108, Anambra State, Nigeria; chukwudiokani@gmail.com

**Keywords:** ESBL, Enterobacteriaceae, gram-negative bacteria, antibiotic resistance, childhood infections

## Abstract

Extended-spectrum β-lactamase (ESBL)-producing organisms have become a serious challenge in healthcare delivery globally. The prevalence of ESBL carriage in healthy and sick children in Enugu, Nigeria, was bacteriologically investigated in this study. Four hundred and twenty-two biological samples (mid-stream urine and feces) were bacteriologically analyzed. The isolates were screened for ESBL production using Clinical and Laboratory Standards Institute (CLSI) breakpoints. The suspected ESBL producers were confirmed using double disc synergy test method. Out of the 162 isolates screened, 32 (19.8%) were confirmed as ESBL positive, with a prevalence of 25.32% among sick children in Enugu State University Teaching Hospital (ESUTH), Parklane, Enugu and 13.89% in apparently healthy children in a community setting. *Klebsiella* spp. and *Escherichia coli* had the highest prevalence of 34.6% and 28.6%, respectively; *Citrobacter* spp. and *Enterobacter* spp. were 18.2% and 16.7%, respectively. The ESBL positive isolates were resistant to sulfamethoxazole/trimethoprim (100%), tetracycline (100%), kanamycin (96.9%), nitrofurantoin (84.4%), ciprofloxacin (68.6%), and chloramphenicol (62.5%) but susceptible to meropenem (100%), colistin (56.3%), and gentamicin (50%). *Klebsiella* spp. had the highest ESBL occurrence among sick children while *E. coli* had the highest ESBL occurrence among healthy children in Enugu. All ESBL-positive isolates were multiply resistant to conventional antibiotics. The emergence and spread of β-lactamase-producing Enterobacteriaceae in hospital and community environments highlight the possibility for an infection outbreak if not checked.

## 1. Introduction

Antimicrobial resistance has been identified as one of the greatest threats to human health, and developing countries like Nigeria are worst hit by this crisis [1]. The major cause of this crisis is the indiscriminate and widespread use of antimicrobial agents [2], especially antibiotics containing a β-lactam ring, in prophylaxis and the treatment of bacterial diseases [3]. The misuse and abuse of β-lactam antibiotics has led to antibiotic selective pressure and the development of resistance to these drugs by most bacteria, particularly the Enterobacteriaceae, of which β-lactamase production remains the most important contributing factor to this resistance [4,5]. Extended-spectrum β-lactamases (ESBLs) have the extended ability to hydrolyze and cause resistance to various types of the newer β-lactam antibiotics, such as the expanded-spectrum cephalosporins and monobactams [6,7]. Some β-lactamases, like CTX-M and PER, are natural ESBLs, while other enzymes, such as TEM and SHV variants, acquire single or multiple amino acid substitutions/changes that extend their spectrum to cephalosporins [6,8]. The rapid emergence of ESBL-producing Enterobacteriaceae has drawn global attention because they are important causative agents of hospital infections typically associated with pneumonia, urinary tract infections, bacteremia, and other intra-abdominal infections [9,10]. The spread of these ESBLs has posed a threat to health, including hindering effective treatment, prolonged hospitalization, and increased treatment costs [10]. These have necessitated the need for extensive peer review research on ESBLs and increased awareness campaign on antimicrobial resistance, most especially in developing countries like Nigeria, where there is no or less surveillance activity and regulations guiding the use of antibiotics. In Nigeria, there are no previous studies and reports on the prevalence of ESBL-producing Enterobacteriaceae (ESBL-PE) in children. This study is imperative as it is poised to provide information on the prevalence of ESBL-PE in both healthy and sick children in Enugu Metropolis and highlights the resistance patterns of these organisms to non-β lactam antibiotics. It also reveals possible risk factors to the spread of these organisms in hospital or community settings and highlights the best-practice infection control measures and appropriate choice of empirical antimicrobial coverage for ESBL infections in this population. This is critical for better defining the prevalence and characterization of risk factors to ESBL-producing Enterobacteriaceae in children, in order to adopt infection control best-practice measures and help in the appropriate choice of empirical antimicrobial coverage for ESBL infections in this population. This study therefore sought to bridge these gaps in knowledge. 

## 2. Materials and Methods 

### 2.1. Patients

A total of 422 clinical samples (mid-stream urine and feces) were collected and this comprised 233 samples from sick children from Enugu State University Teaching Hospital (ESUTH) and 105 samples from healthy school children in Abakpa and 84 samples from healthy school children in Emene, after permission had been sought for and obtained from the authorities of the schools and informed consent obtained from the guardians of these children. The study protocol was approved by the Ethical Committee of ESUTH (Approval number: ESUTHP/C-MAC/RA/034/177) and signed on 24 March 2017

### 2.2. Isolation 

The clinical specimens were inoculated on MacConkey agar plates and incubated at 37 °C for 24 h. Biochemical characterization of the isolates was achieved by subjecting them to biochemical tests for identification and differentiation of members of the Enterobacteriaceae according to the method of Tille [11].

### 2.3. ESBL Screening Test

ESBL screening test of the isolates were determined using the Kirby–Bauer disc diffusion method according to The Clinical & Laboratory Standards Institute (CLSI) performance standards for antimicrobial disc susceptibility tests [12] using aztreonam (30 µg), cefepime (30 µg), cefotaxime (30 µg), cefoxitin (30 µg), ceftazidime (30 µg), and ceftriaxone (30 µg).

### 2.4. Double Disc Synergy Test

All isolates that were resistant to any of the β-lactam antibiotics were screened for ESBL production using the Double Disc Synergy Test (DDST). Suspensions of the isolates equivalent to 0.5 McFarland equivalent standards were streaked on the surface of a sterile Mueller Hinton agar plates, according to CLSI performance standards for antimicrobial disc susceptibility tests [12]. After 25 min of pre-incubation, combination discs of 20 µg amoxicillin/10 µg clavulanic acid (Oxoid, Wade Road, Basingstoke, Hampshire, RG24 8PW, UK) were be placed at the center of the plates and 30 µg ceftazidime/30 µg cefotaxime discs (Oxoid, UK) were placed 15 mm apart from the center discs and incubated at 37 °C for 18 h. The isolates, whose zone around the test antibiotic discs increased towards the center disc (amoxicillin 20 µg/clavulanic acid 10 µg), were considered to be positive for ESBL production. 

### 2.5. Susceptibility Test of Isolates to Non-β-Lactam Antibiotics 

The susceptibility of the ESBL positive isolates to non-β lactam antibiotics from different classes of antibiotics were determined using the Kirby–Bauer disc diffusion method, according to CLSI performance standards for antimicrobial disc susceptibility tests [12]. The isolates were challenged with the following different classes of antibiotics (Oxoid, UK): amikacin (30 µg), chloramphenicol (30 µg), ciprofloxacin (5 µg), colistin sulfate (25 µg), gentamicin (30 µg), kanamycin (5 µg), meropenem (10 µg), nitrofurantoin (300 µg), tetracycline (30 µg), and sulfamethoxazole/trimethoprim (25 µg). Their inhibitory zone diameters were interpreted as susceptible (S), intermediate (I), or resistant (R), according to the zone diameter breakpoints of the performance standards of antibiotic susceptibility testing of CLSI [12].

### 2.6. Multiple Antibiotic Resistance Index 

The multiple antibiotic resistance index is calculated as the ratio of the number of antibiotics to which the isolates were resistant to the total number of antibiotics against which the isolates were tested (a/b). Multiple antibiotic resistance index (MARI) values greater than 0.2 (20%) were considered a high-risk source of contamination where antibiotics are often used, as described in previous reports [13,14].

### 2.7. Statistical Data Analysis

Data analysis was conducted using GraphPad Prism version 5.00 for Windows (GraphPad Software, Inc., San Diego, CA, USA). Descriptive data analysis of the characteristics of the study participant was conducted to evaluate the characteristics of the study participants in relation to ESBL-PE carriage rate or colonization frequency. Chi-square test was used to describe the carriage of Enterobacteriaceae in the urine and fecal samples of the children in the hospital and community setting and in highlighting possible risk factors. 

## 3. Results

A total of 422 biological samples were collected out of which 233 (55.21%) came from ESUTH (hospital environment) while 189 (44.79%) came from community (Table 1). 

Of the 233 samples from ESUTH, 79 (33.91%) were positive for Enterobacteriaceae in the hospital while out of the 189 samples from community setting, 108 (57.14%) had Enterobacteriaceae carriage either in their urine of feces (Table 2).

From Table 2 also, the chi-square test for carriage of Enterobacteriaceae in the urine and fecal samples of the children in the hospital and community setting revealed a significant difference, with a *p*-value of 0.0033 and chi-square, degrees of freedom (df) = 17.73, 5. With a total of 79 Enterobacteriaceae isolates in a hospital setting, the prevalence of ESBL-producing Enterobacteriaceae isolates was 25.32%, while in the community setting, the prevalence was much lower (13.89%). The chi-square test for trend (chi-square, df = 0.02790, 1) showed that there were equal chances of occurrence of ESBL and non-ESBL isolates in the hospital (*p* = 0.8674). The same was also true in community setting (chi-square, df = 0.003946, 1 and *p* = 0.9499). The chi-square for trend for ESBL positivity in Hospital versus Community (chi-square, df = 3.273, 1) showed no significant difference (*p* = 0.0704). This implies that the trend of ESBL-producing Enterobacteriaceae is equal in both settings. 

In ESUTH, a total of 233 sick children participated in the study (Table 3), of which 114 were males and 119 were females. As many as 20 children representing 8.58% were ESBL-PE carriers. Males had a higher ESBL-PE colonization frequency than females, although this was not significantly different (*p* > 0.05). Chi-square analysis of the risk factors associated with Enterobacteriaceae carriage among the sick children in ESUTH showed that of all the possible risk factors/covariates considered, the source of drinking water and non-practice of regular hand washing were the only factors associated with the infection with *p* < 0.0001 and *p* = 0.0067, respectively. Considering ESBL-PE carriage no risk factors were found.

In the community setting (Table 4), the risk factors associated with Enterobacteriaceae carriage included stage in school (daycare, nursery, and primary) with *p* = 0.0128, source of drinking water with *p* = 0.0001, and school type (public versus private schools) with *p* = 0.0004. The risk factors for ESBL-PE carriage were indeterminate. The bivariate analyses of the covariates in Enterobacteriaceae and ESBL-PE carriage in sick and healthy children revealed no significant risk.

The ESBL-producing Enterobacteriaceae (ESBL-PE) isolates showed high resistance (Table 5) to tetracycline, co-trimoxazole, nitrofurantoin, and kanamycin. However, they showed average susceptibility to colistin and gentamicin. The 100% susceptibility of the ESBL-PE isolates to meropenem was noted in this study. Two-way ANOVA of the isolate susceptibilities to the used drugs showed that they were significantly resistant (*p* = 0.0163). Also, all the ESBL-PE isolates had a MARI greater than 0.2 (20%) giving an incidence of multi-antibiotic resistance strains of 100% (Table 6).

## 4. Discussion

Several studies have demonstrated the prevalence of ESBL-producing Enterobacteriaceae in many parts of the world [15,16], and a number of individual reports are available in Nigeria [17,18,19]. This study was undertaken to bridge the gap on the paucity of data on the prevalence of ESBL-producing Enterobacteriaceae in a purely pediatric population in this part of the world. The overall prevalence of ESBL-PE among sick children in ESUTH was 8.58%, whereas in healthy children, it was 6.34%. This shows that the ESBL-PE carriage rate was slightly higher among the sick children compared to the healthy children, and this may be as a result of higher prevalence and dissemination of ESBL pathogens in hospital settings as reported in various studies [17,20,21]. The overall ESBL-PE prevalence of 6.34% among healthy children in community setting is lower than what was reported in healthy community Tanzanian (11.2%) and Libyan children 13.4% [22,23]. However, our report showed a higher than prevalence in healthy Portuguese (2.7%), Swedish (2.9%), French (4.6%) children [24]. Our report also showed much lower prevalence than that of 23% in Lao preschool children, 24.8% in healthy Lebanese children, and 59% in healthy Banguian children, Central African Republic [25,26,27]. The observed 8.58% prevalence of ESBL-PE among sick children in ESUTH is much lower when compared to the prevalence of 31% found in hospitalized children in Niger and 32.6% recorded in Guinea-Bissau [28,29]. Out of all the ESBL-PE isolates from sick children in ESUTH, *Klebsiella* spp. and *Escherichia coli* had the highest prevalence of 34.6% and 28.6% respectively, compared to *Citrobacter* spp. with 18.2% and *Enterobacter* spp. with 16.7%. Although, it has been said to be difficult to make valid comparison of the prevalence of ESBLs because of the variation in study design [30], the findings from this study are comparable to the work of Yahaya et al. [31], which recorded an ESBL prevalence of 23.8% and 30% for *E. coli* and *Klebsiella* spp., respectively, among sick individuals in Maiduguri Teaching Hospital, Nigeria. Previous studies from Nigeria, although not specifically focused on children, have reported ESBL production in *Klebsiella* spp., *E. coli*, *Enterobacter* spp., and *Citrobacter* spp. from hospital settings and from 2003 to 2017, the rate varied from 6% to 87% [17,32,33,34]. The high ESBL prevalence detected in *Klebsiella* spp. compared to *Escherichia coli* is in agreement with the findings of some of these studies in Nigeria. The reason for high ESBL prevalence in *Klebsiella* spp. could be connected with the fact that *Klebsiella* spp. tend to be more associated with nosocomial infections than *E. coli*, hence it has more chance to acquire multidrug resistance plasmids and disseminate more in the hospital settings [35,36]. Our findings showed that all the ESBL-PE isolates were resistant to multiple antibiotics, suggesting an incidence of multi-antibiotic resistance strains of 100%. This points to poor compliance to antibiotic policy, high use or misuse of antibiotics, high rate of transfer of multi-drug resistant plasmids from species to species, clonal dissemination of ESBL-PE from patients to patients in the hospital settings and to healthy individuals in the community (that serve as reservoirs of these pathogens) and emerging of new strains of community-acquired ESBL-producing *Enterobacteriaceae*. Our study showed that colistin resistance was as high as 43.75%, contrary to known resistance rates to polymyxins, which are generally between 10% and 23% [37,38]. Colistin-resistant organisms had been postulated to spread from animals to human [39,40,41,42], suggesting the concept of one health. Observational studies have shown that polymyxins are used profusely in animal feed in the community to enhance growth. The observed high resistance to colistin in our study calls for caution and regulation in such unlicensed/off-label use of antibiotics. Colistin resistance among ESBL-producing Enterobacteriaceae is becoming a public health concern and is postulated to be due to the plasmid-borne *mcr-1* gene [38,42,43].

Therefore, for effective treatment of ESBL infections, empirical antibiotic regimens should be selected based on individual antibiogram. There is need for a multifactional approach combining continued research, prudent use of antibiotics, effective ESBL infections control measures and rapid detection of ESBL-producing organisms in routine clinical laboratory in order to contain the emergence and spread of ESBL-producing Enterobacteriaceae in the human population.

The source of drinking water, non-practice of regular hand washing, the stage in school (daycare, nursery, and primary), and school type (public versus private schools) were the common factors associated with Enterobacteriaceae infections in this study. Previous studies [44,45] have observed that demographic variables and comorbid conditions predisposed patients to carbapenemase-producing Enterobacteriaceae infections. Also, clean water has been claimed to be the most important public health intervention strategy, followed by vaccination, in promoting both individual and global health [46,47]. This study therefore calls for the need to control infectious diseases through the regular and timely provision of clean drinking water. Also important in infection control is the continuous and consistent adoption of hygienic practices [48] and mass education through all available public and social media as well as regular hand washing.

## 5. Conclusions

The prevalence of ESBL-producing bacteria was higher in sick children compared to healthy children in Enugu. *Klebsiella* spp. had the highest ESBL occurrence among sick children while *E. coli* had the highest ESBL occurrence among healthy children in Enugu. All the ESBL positive isolates were multiply resistant to conventional antibiotics. Meropenem proved to be the drug of choice in the management of infections caused by isolates in both community and hospital settings.

## Figures and Tables

**Table 1:** The distribution of samples used for this study.

Setting	Location	Number of Specimen Used		
		Urine, *N* (%)	Feces, *N* (%)	Total, *N* (%)
Hospital	ESUTH (sick children)	128 (30%)	105 (25%)	233 (55%)
Community	Abakpa (apparently healthy children)	58 (14%)	47 (11%)	105 (25%)
	Emene (apparently healthy children)	46 (11%)	38 (9%)	84 (20%)

Note: ESUTH = Enugu State University Teaching Hospital.

**Table 2:** Extended-spectrum β-lactamase (ESBL) confirmatory testing of the Enterobacteriaceae isolates.

Enterobacteriaceae Isolates	ESUTH (Hospital Setting)			Abakpa/Emene (Community Setting)		
	Positive for ESBL	Negative for ESBL	Total	Positive for ESBL	Negative for ESBL	Total
*Citrobacter* spp.	2	9	11	0	12	12
*Enterobacter* spp.	1	5	6	0	4	4
*Escherichia coli*	8	20	28	8	28	36
*Klebsiella* spp.	9	15	24	4	17	21
*Proteus* spp.	0	7	7	0	7	7
*Serratia* spp.	0	3	3	3	25	28
Total	20	59	79	15	93	108

Note: ESBL = Extended-spectrum β-lactamase.

**Table 3:** Survey of risk factors associated with ESBL-producing Enterobacteriaceae (ESBL-PE) carriage in sick children in ESUTH.

Covariates (ESBL-NPE = ESBL Non-Producing Enterobacteriaceae)		Prevalence					
		Total Sampled *N* (%) 233 (100%)	Enterobacteriaceae Positive *N* (%) 81 (34.76)	Enterobacteriaceae Negative *N* (%) 152 (65.24)	ESBL-PE *N* (%) 20 (8.58)	ESBL-NPE *N* (%) 61 (26.18)	ESBL-PE Colonization Frequency %
Age (years)	0–5	101 (43.35)	34 (33.66)	67 (66.34)	6 (5.94)	28 (27.72)	17.6% (6/34)
	6–10	88 (37.77)	28 (31.82)	60 (68.18)	10 (11.36)	18 (20.45)	35.7% (10/28)
	11–15	44 (18.88)	19 (43.18)	25 (56.82)	4 (9.09)	15 (34.09)	21.1% (4/19)
Gender	Male	114 (48.93)	39 (34.21)	75 (65.79)	11 (9.65)	28 (24.56)	28.2% (11/39)
	Female	119 (51.07)	42 (35.29)	77 (64.71)	9 (7.56)	33 (27.73)	21.4% (9/42)
Type of school	Private	136 (58.37)	30 (22.06)	106 (77.94)	5 (3.68)	25 (18.38)	16.7% (5/30)
	Public	97 (41.63)	51 (52.58)	46 (47.42)	15 (15.46)	37 (38.144)	29.4% (15/51)
Stage in school	Daycare	35 (15.02)	15 (42.86)	20 (57.14)	2 (5.71)	13 (37.14)	13.3% (2/15)
	Nursery	68 (29.18)	40 (58.82)	28 (41.18)	10 (14.71)	30 (44.12)	25% (10/40)
	Primary	130 (55.79)	26 (20.00)	104 (80.00)	8 (6.15)	18 (13.85)	30.8% (8/26)
Who normally prescribed antibiotics given to the child?	Doctors	63 (27.03)	22 (34.92)	41 (65.08)	3 (4.76)	19 (30.16)	13.6% (3/22)
	Pharmacists	25 (10.73)	12 (48.00)	13 (52.00)	1 (4.00)	11 (44.00)	8.3% (1/12)
	PPMD*	145 (62.23)	47 (32.41)	98 (67.59)	16 (11.03)	31 (21.38)	38.1% (16/42)
Hospital admission in the last 3–6 months	Yes	77 (33.04)	33 (42.86)	44 (57.14)	15 (19.48)	18 (23.38)	45.5% (15/33)
	No	156 (66.95)	48 (30.77)	108 (69.23)	5 (3.21)	43 (27.56)	10.4% (5/48)
Share public toilet at home	Yes	48 (20.60)	21 (43.75)	27 (56.25)	3 (6.25)	18 (37.50)	31.4% (11/35)
	No	36 (15.45)	6 (16.67)	30 (83.33)	0 (0.00)	6 (16.67)	19.7% (9/46)
Practice of regular hand washing	Yes	85 (36.48)	20 (23.53)	65 (76.47)	3 (3.53)	17 (20.00)	15% (3/20)
	No	148 (63.52)	61 (41.22)	87 (58.78)	17 (11.49)	43 (29.05)	27.9% (17/61)
Source of water supply at home	Tap	84 (86.05)	9 (10.71)	75 (89.29)	1 (1.19)	8 (9.52)	11.1% (1/9)
	Well	117 (50.21)	53 (45.30)	64 (54.70)	16 (13.68)	37 (31.62)	30.2% (16/53)
	Bore-hole	32 (13.73)	19 (59.38)	13 (40.63)	3 (9.38)	16 (50.00)	15.8% (3/19)
	River	0 (0.00)	0 (0.00)	0 (0.00)	0 (0.00)	0 (0.00)	0.0%
Type of nutrition	Breast milk	29 (12.45)	8 (27.59)	21 (72.41)	0 (0.00)	8 (27.59)	0.0%
	Dairy food	37 (15.88)	19 (51.35)	18 (48.65)	7 (18.92)	12 (32.43)	36.8% (7/19)
	Household food	167 (71.67)	54 (32.34)	113 (67.66)	13 (7.78)	41 (24.55)	24.1% (13/54)

PPMD* = Patent and Proprietary Medicine Dealer. They are also called private patent medicine vendors. They include individuals not possessing any degree in pharmacy but sell standard health care pharmaceutical and medical products to the public for the purpose of making profit.

**Table 4:** Survey of risk factors associated with ESBL-PE carriage among the apparently healthy children.

Covariates [ESBL-NPE = ESBL Non-Producing Enterobacteriaceae]		Prevalence					
		Total Sampled *N* (%) 189 (100%)	Enterobacteriaceae Positive *N* (%) 81 (42.86)	Enterobacteriaceae Negative N (%) 108 (57.14)	ESBL-PE *N* (%) 12 (6.34)	ESBL-NPE *N* (%) 69 (75.31)	ESBL-PE Colonization Frequency (6.88%)
Age (years)	0–5	51 (26.98)	28 (54.90)	23 (45.10)	3 (10.71)	25 (89.29)	5.88
	6–10	102 (53.96)	39 (20.63)	63 (61.76)	7 (17.95)	32 (82.05)	6.86
	11–15	36 (19.05)	14 (7.41)	22 (61.11)	2 (14.29)	12 (85.71)	5.56
Gender	Male	81 (42.86)	38 (20.11)	43 (53.09)	7 (18.42)	31 (81.58)	8.64
	Female	108 (57.14)	43 (22.75)	65 (60.19)	5 (11.63)	38 (88.37)	4.63
Type of school	Private	99 (52.38)	30 (15.87)	69 (69.70)	3 (10.00)	27 (90.00)	3.03
	Public	90 (47.62)	51 (26.98)	39 (43.33)	9 (17.65)	42 (82.35)	10.00
Stage in school	Daycare	34 (17.99)	7 (3.70)	27 (79.41)	0 (0.00)	7 (100.00)	0.00
	Nursery	51 (26.98)	26 (13.76)	25 (49.02)	3 (11.54)	23 (88.46)	5.88
	Primary	104 (55.03)	48 (25.40)	56 (53.85)	9 (18.75)	39 (81.25)	8.65
Who normally prescribed antibiotics given to the child?	Doctors	30 (15.87)	5 (2.65)	25 (83.33)	0 (0.00)	5 (100.00)	0.00
	Pharmacists	14 (7.41)	3 (1.59)	11 (78.57)	0 (0.00)	3 (100.00)	0.00
	PPMD	145 (76.72)	73 (38.64)	72 (49.66)	12 (16.44)	61 (84.62)	8.28
Hospital admission in the last 3–6 months	Yes	36 (19.05)	13 (6.89)	23 (63.89)	1 (7.69)	11 (84.62)	2.78
	No	153 (80.95)	68 (35.98)	85 (55.56)	11 (16.18)	57 (83.82)	7.19
Share public toilet at home	Yes	89 (47.09)	50 (26.46)	39 (43.82)	9 (18.00)	41 (82.00)	10.11
	No	100 (52.91)	31 (16.40)	69 (69.00)	3 (9.68)	28 (90.32)	3.00
Practice of regular hand washing	Yes	48 (25.40)	12 (6.35)	36 (75.00)	1 (8.33)	11 (91.67)	2.08
	No	141 (74.60)	69 (36.51)	72 (51.06)	11 (15.94)	58 (84.06)	7.80
Source of water supply at home	Tap	27 (14.29)	2 (1.06)	25 (92.59)	0 (0.00)	2 (100.00)	0.00
	Well	142 (75.13)	72 (38.10)	70 (49.30)	12 (16.67)	60 (83.33)	8.45
	Bore-hole	20 (10.58)	7 (3.70)	13 (65.00)	0 (0.00)	7 (100.00)	0.00
	River	0 (0.00)	0 (0.00)	0 (0.00)	0 (0.00)	0 (0.00)	0.00
Type of nutrition	Breast milk	0 (0.00)	0 (0.00)	0 (0.00)	0 (0.00)	0 (0.00)	0.00
	Dairy food	24 (12.70)	5 (2.65)	19 (79.17)	2 (40.00)	3 (60.00)	8.33
	Household food	165 (87.30)	76 (40.21)	89 (53.94)	10 (13.16)	66 (86.84)	6.06

**Table 5:** Overall antibiogram of all the ESBL-PE isolates.

Antibiotics	Susceptible	Intermediate Susceptible	Resistant	Total
Meropenem (10 µg)	32	0	0	32
Amikacin (30 µg)	11	19	2	32
Colistin Sulfate (25µg)	18	0	14	32
Gentamicin (30 µg)	16	1	15	32
Chloramphenicol (30 µg)	10	2	20	32
Ciprofloxacin (5 µg)	7	3	22	32
Nitrofurantoin (300 µg)	5	0	27	32
Kanamycin (5 µg)	0	1	31	32
Tetracycline (30 µg)	0	0	32	32
Co-trimoxazole (25 µg)	0	0	32	32

**Table 6:** Overall multi-antibiotic resistance index (MARI) of all the ESBL-producing Enterobacteriaceae (ESBL-PE) isolates.

Setting	From Sick Children			From Healthy Children		
Isolates Number	Number of Antibiotics Isolates Were Resistant to (a)	MARI (a/b)	% MARI	Number of Antibiotics Isolates Were Resistant to	MARI (a/b)	% MARI
***Citrobacter* spp. (*n* = 2)**						
**1**	7	0.70	70			
**2**	6	0.60	60			
***Enterobacter* spp. (*n* = 1)**						
**1**	5	0.5	50			
***Escherichia coli* (*n* = 8)**				***Escherichia coli* (*n* = 8)**		
**1**	7	0.70	70	4	0.40	40
**2**	7	0.70	70	5	0.50	50
**3**	3	0.30	30	6	0.60	60
**4**	8	0.80	80	4	0.40	40
**5**	7	0.70	70	7	0.70	70
**6**	6	0.60	60	6	0.60	60
**7**	6	0.60	60	7	0.70	70
**8**	5	0.50	50	7	0.70	70
***Klebsiella* spp. (*n* = 9)**				***Klebsiella* spp. (*n* = 4)**		
**1**	8	0.80	80	5	0.50	50
**2**	6	0.60	60	6	0.60	60
**3**	6	0.60	60	7	0.70	70
**4**	7	0.70	70	5	0.50	50
**5**	8	0.80	80			
**6**	4	0.40	40			
**7**	7	0.70	70			
**8**	6	0.60	60			
**9**	6	0.60	60			

Note: (a) = Number of Antibiotics Isolates were Resistant to while (b) = Total Number of Antibiotics Used/tested = 10.
